# Cancer Genome Sequencing and Its Implications for Personalized Cancer Vaccines

**DOI:** 10.3390/cancers3044191

**Published:** 2011-11-25

**Authors:** Lijin Li, Peter Goedegebuure, Elaine R. Mardis, Matthew J.C. Ellis, Xiuli Zhang, John M. Herndon, Timothy P. Fleming, Beatriz M. Carreno, Ted H. Hansen, William E. Gillanders

**Affiliations:** 1 Department of Surgery, Washington University School of Medicine, St. Louis, MO 63110, USA; E-Mails: lilij@wudosis.wustl.edu (L.L.); goedegep@wudosis.wustl.edu (P.G.); zhangxiu@wudosis.wustl.edu (X.Z.); herndonj@wudosis.wustl.edu (J.M.H.); flemingt@wudosis.wustl.edu (T.P.F.); 2 The Alvin J. Siteman Cancer Center at Barnes-Jewish Hospital and Washington University School of Medicine, St. Louis, MO 63110, USA; E-Mails: emardis@wustl.edu (E.R.M.); mellis@dom.wustl.edu (M.J.C.E.); bcarreno@dom.wustl.edu (B.M.C.); hansen@pathology.wustl.edu (T.H.H.); 3 The Genome Institute at Washington University School of Medicine, St. Louis, MO 63108, USA; 4 Department of Medicine, Washington University School of Medicine, St. Louis, MO 63110, USA; 5 Department of Pathology and Immunology, Washington University School of Medicine, St. Louis, MO 63110, USA

**Keywords:** cancer genome sequencing, unique tumor antigen, DNA vaccine

## Abstract

New DNA sequencing platforms have revolutionized human genome sequencing. The dramatic advances in genome sequencing technologies predict that the $1,000 genome will become a reality within the next few years. Applied to cancer, the availability of cancer genome sequences permits real-time decision-making with the potential to affect diagnosis, prognosis, and treatment, and has opened the door towards personalized medicine. A promising strategy is the identification of mutated tumor antigens, and the design of personalized cancer vaccines. Supporting this notion are preliminary analyses of the epitope landscape in breast cancer suggesting that individual tumors express significant numbers of novel antigens to the immune system that can be specifically targeted through cancer vaccines.

## 1. Introduction

In 2001, two research groups reported the completion of the first draft assemblies of the human genome sequence [1,2]. Ten years after this milestone achievement, massively parallel DNA sequencing (so-called “next-generation”) technologies have transformed genome sequencing, significantly, decreasing both the cost and time required to sequence human genomes, and by extension cancer genomes. However, the overall impact of cancer genome sequencing on human health has not been fully realized. In this review, we discuss the evolution of cancer genome sequencing and its potential application towards the development of personalized cancer vaccines.

## 2. Evolution of DNA Sequencing Technologies

DNA sequencing technology has made great advances over the past 30 years since the development of the chain-terminating “Sanger method” [3,4]. The first draft of the human genome [1,2] was produced largely by Sanger-based capillary electrophoresis technology. Sequencing the entire genomes of organisms using the Sanger method has proven to be difficult and time consuming. Through significant refinements and automation, the current Sanger method-based instruments are able to deliver read lengths up to 1,000 bases and allow 384 samples to be sequenced in parallel within a few hours. Therefore, even using the most advanced Sanger sequencers, it would take years and millions of dollars to sequence a human genome.

**Figure 1.**
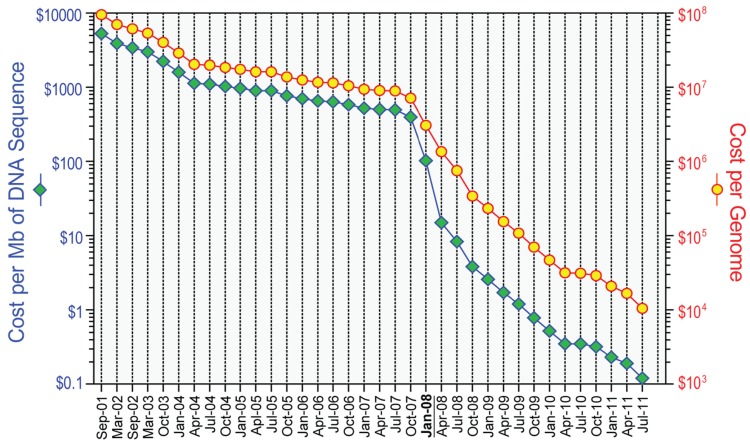
The plummeting cost of genome sequencing. The cost-accounting data, available at the website of National Human Genome Research Institute (NHGRI) [12], are summarized relative to two metrics: (1) the cost of determining one megabase (10^6^ bases) of DNA sequence of a specified quality [12], and (2) the cost of sequencing a human-sized genome (*i.e.*, 3,000 Mb). Of note, the sudden and profound decrease beginning in January 2008 represents the time when the NHGRI sequencing centers transitioned from Sanger-based chemistry and capillary-based instruments to next-generation DNA sequencing technologies.

However, the field has changed rapidly since the commercial introduction of several “massively parallel” or next-generation platforms, starting in 2004. The technical details of the next-generation DNA sequencing technologies are beyond the scope of this review but have been described elsewhere [3-6]. In general, next-generation sequencing platforms produce shorter sequencing reads with slightly lower per base accuracy than data from Sanger-based DNA sequencing, and as such require increased coverage depths. These shorter read depths also complicate read-based assembly as a means of genome sequencing for more complex genomes (such as human). As such, all human genome sequencing with next-generation methods relies on alignment of the sequencing reads to the human reference sequence as a precursor to identifying mutations or other genomic alterations. Thanks to the ever-growing computing power and advances in instrument design, recent years have seen major increases in speed (Table 1) and reduction in cost (Figure 1) for next-generation methods, although the cost of analysis of next-generation sequencing data has not decreased as dramatically.

The future of DNA sequencing looks even more promising as new technologies continue to emerge (Table 1). For example, Complete Genomics has developed a “DNA nanoball” sequencing technology that uses fluorescent probe ligation chemistry similar to the SOLiD platforms. However, instead of an emulsion PCR step, the method uses rolling circle replication to amplify small DNA fragments into “DNA nanoballs” [7,8]. In a pH-based sensing system similar to the Roche/454 pyrosequencing technology, Ion Torrent's Personal Genome Machine is a simple, scalable and fast machine that “reads” DNA without requiring optical detection [9]. The Helicos' system generates sequence information by capturing images of fluorescent step-wise DNA synthesis reactions from individual molecules, without prior DNA amplification [10]. This approach avoids sequencing errors attributable to PCR artifacts, but has an inherently higher error rate than amplified DNA technologies, due to noise-related artifacts that are unique to single-molecule sequencing. In 2010, Pacific Biosciences introduced its “third generation” sequencing product, the PacBio *RS* which also is a single molecule sequencing platform. The RS uses nanofabricated structures called “zero mode waveguides” or ZMWs to focus the instrument optics on individual DNA polymerases as they copy single molecules of DNA by incorporating fluorescent nucleotides. These real-time movies of DNA polymerization can result in read lengths in excess of 1,500 base pairs, although the error rate also is higher as a result of single-molecule detection sources of noise [11]. Both the Ion Torrent and Pacific Biosciences instruments have lower yields of sequencing data per run than other massively parallel sequencing instruments at this time, and are not suited for whole human genome sequencing. These new technologies may further reduce time and/or the cost of genome sequencing.

## 3. Cancer Genome Sequencing

**Figure 2.**
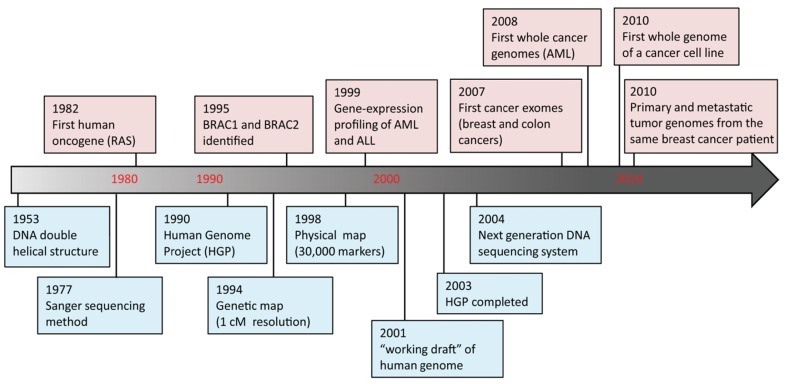
Human cancer genome timeline. Selected milestone events [13-16,18-23] are illustrated for human genome sequencing (blue boxes) and cancer genomics (pink boxes). Decade marks in red are not drawn to scale.

DNA sequencing advances are making large-scale personal genome sequencing a rapidly approaching reality. Cancer genomics is probably the field that has been impacted most profoundly. In the past few years, there have been a number of diverse publications on cancer genomic studies [13-16]. Some studies focused on examination of all exons (exome) in a few cancers; others examined hundreds of genes in hundreds of samples, while whole genome sequencing of a cancer cell line or a matched tumor/normal pair has been performed as well (Figure 2). Genome instability and mutations have been established as an “enabling characteristic” of cancer [17]. Genetic alterations are not only considered to be the direct cause of cancers but also, in some cases, of their progression as tumors develop and metastasize. Since both tumor tissue and normal specimens can be obtained from the same patient, and most genetic alterations present in cancers represent somatic events, changes identified through cancer genome sequencing are most likely to be cancer-specific.

Despite the valuable insights gained so far from cancer genome sequencing, the results highlight the complexity of this disease. An immediate challenge is to translate insights from cancer genome sequencing into the clinic. Two recent studies highlight the power of cancer genome sequencing as a diagnostic tool, helping doctors make “real-time” decisions. In one case, whole-genome sequencing of a 39-year-old woman with acute myeloid leukemia (AML) revealed a novel insertional translocation on chromosome 17 that resulted in a pathogenic bcr3 *PML-RARA* fusion gene [24]. The genomic data were obtained within seven weeks and led to a change in treatment. Instead of a stem cell transplant, the patient received targeted chemotherapy with retinoic acid and is now in remission [24]. In the second study, genome sequencing on skin and leukemia cells from a woman who died at age 42 after developing breast and ovarian cancer and then leukemia allowed researchers to identify a novel deletion of 3 exons of the *TP53* gene [25]. Although the discovery did not save the patient's life, genetic counseling and testing were recommended for her three children, who are at high risk of developing cancer at a young age if they inherited this mutation. In both cases, these “cryptic” genetic events would not have been detected with classic cytogenetic techniques.

## 4. Unique Tumor Antigens

There is overwhelming evidence of a dynamic crosstalk between the immune system and cancer. The current concept of “cancer immunoediting” recognizes the dual roles of the immune system: host-protection and tumor-selection [26]. Cancer vaccines are designed to stimulate or restore the immune system's natural ability to recognize and destroy tumors. Two reviews were recently published and offer an in-depth overview of cancer vaccines [27,28]. Preventive vaccines against cancer-causing pathogens (e.g., hepatitis B virus and human papillomavirus) have reduced the risks of developing certain cancers, namely, hepatocarcinoma and cervical cancer, through induction of protective immune responses to the pathogens. However, the development of therapeutic cancer vaccines has been disappointing in the past. Recent progress in the identification of tumor antigens, and in the elucidation of antigen presentation pathways and effector mechanisms has renewed interest in vaccine therapy for cancer. The current paradigm in cancer vaccine development is to target shared tumor antigens. We propose an innovative new paradigm: development of personalized cancer vaccines targeting unique tumor antigens identified by genome sequencing. Next-generation DNA sequencing technologies have fundamentally transformed cancer genome sequencing, as outlined above. These innovative technologies also provide an unprecedented opportunity to rapidly identify unique tumor antigens toward the goal of a personalized cancer vaccine for each patient.

### 4.1. What is a Unique Tumor Antigen?

Tumor antigens are often classified as “unique” or “shared” tumor antigens, according to their distribution in normal or in neoplastic tissues (Table 2). Shared tumor antigens are expressed in multiple cancers, and are often self-differentiation antigens that are expressed in a limited subset of normal tissues, but overexpressed in cancers. Examples of shared tumor antigens include MAGE (melanoma) [29], prostatic acid phosphatase (prostate cancer) [30], and HER2/neu (breast cancer) [31]. They also include mutated oncogenes or tumor suppressor genes, or chromosomal translocations encoding novel fusion proteins such as K-Ras, p53, and BCR-ABL, respectively.

Unique tumor antigens are only expressed in a single cancer, and are typically the result of point mutations or other genetic changes present only in the tumor [32,33]. As such, unique tumor antigens represent the only true tumor-specific antigens that are not expressed in any normal tissues. In mice, unique tumor antigens were described many years ago in models of chemical or UV-induced fibrosarcomas. These tumors were rejected through T cell-mediated immunity upon transplantation in syngeneic hosts. The immune response was found to be unique to each tumor which led to the concept that such tumors express unique tumor-specific transplantation antigens [32,33]. In 1995, the first unique tumor antigens in humans were identified in melanoma [34,35]. Since that time additional publications have described the expression of unique tumor antigens in melanoma [36], non-small cell lung cancer [37] and other human cancers [33,38]. Not surprisingly, unique antigens are typically encoded by genes that regulate cellular processes such as cell cycle, metabolic pathways, and others that are hallmarks of tumor development and survival [17].

To date, no unique tumor antigens have been identified in breast cancer. Current experimental techniques are biased towards the identification of shared tumor antigens such as HER2/neu [39,40], MUC1 [41], and mammaglobin-A [42-44], and no experimental techniques are capable of rapidly or systematically identifying unique tumor antigens. Next-generation DNA sequencing technologies provide an unprecedented opportunity to identify unique tumor antigens. Key questions to be addressed in this evolving paradigm are: (1) Are unique tumor antigens identified by genome sequencing processed and presented by the immune system? (2) Can CD8^+^ T cells recognize these unique tumor antigens? (3) Is there evidence of a pre-existing immune response to unique tumor antigens in breast cancer patients?

### 4.2. Targeting Unique Tumor Antigens: Conceptual Advantages

Once shared tumor antigens were discovered, there was a race to translate the use of these antigens into the clinic, and the majority of cancer vaccine trials performed to date have targeted shared tumor antigens. Unfortunately, the results of these studies have been disappointing [45,46], despite the ability of some vaccines to generate a high frequency of self/tumor antigen-specific T cells [47]. It is now known that the T cell repertoire is edited during development to minimize autoimmunity, and that T cells specific to shared tumor antigens can display low affinity for antigen. This may partially explain the low success rate of cancer vaccines to date, as low affinity T cells are weak effectors, and also are known to promote the expansion of regulatory T cells [48]. Thus, identification of unique tumor antigens not edited by the immune system may elicit more effective T cell responses with increased effector functions.

Cancer vaccine strategies targeting unique tumor antigens have substantial advantages over strategies targeting shared tumor antigens [32,33] (Table 2). (1) Targeting unique tumor antigens is safer. Unique tumor antigens are expressed only in the tumor, decreasing the risk of autoimmunity; (2) Targeting unique tumor antigens is more effective, because T cell responses to unique tumor antigens are high in affinity, and are not limited by mechanisms of self-tolerance; (3) Targeting unique tumor antigens may limit antigen-loss, a common tumor escape mechanism. One of the hallmarks of cancer is genome instability, which may cause the loss of antigen expression on the tumor and thereby recognition by immune cells. As such, one clear weakness of cancer vaccines that target a single shared tumor antigen is antigen-loss. An unbiased strategy, in which all unique tumor antigens are targeted by a vaccine may circumvent antigen-loss and preclude immune escape. In addition, many unique tumor antigens play a functional role in neoplastic transformation (so-called driver mutations). Immune selection resulting in loss of driver mutations may fundamentally alter the phenotype of targeted cancers; (4) Finally, targeting unique tumor antigens is universally applicable. For example, all histologic types of cancer appear to have remarkable numbers of candidate unique tumor antigens, suggesting that a personalized vaccine approach could be applied to treat all types of cancer, regardless of intrinsic subtype.

### 4.3. Targeting Unique Tumor Antigens: Experimental Data

Experiments with mouse antigens have revealed two mechanisms whereby genetic mutations can generate antigenic peptides [29-31,34]. In one scenario, the mutation transforms a non-MHC binding peptide into one that has a high binding affinity for a particular MHC class I allele. Alternatively, the mutation can alter peptide residues that interact with the T cell receptor (TCR) on T cells. These mutated peptides can activate T cells with a TCR repertoire distinct from those that recognize the self/non-mutated peptide.

Results from various clinical studies have indicated patients undergoing immune therapy can develop responses to unique tumor antigens. For example, T cells specific for unique antigens have been identified in clinically responding melanoma patients treated with adoptively transferred T cells [33]. Similarly, patients with metastatic renal cell carcinoma developed specific T cells to mutated peptides encoded by the von Hippel-Lindau (VHL) gene after peptide vaccination [49]. Somatic mutations in VHL have been observed in over 60% of patients with sporadic renal cell carcinoma.

The most compelling evidence to date of the immunogenicity of unique tumor antigens comes from human clinical trials in patients with follicular lymphoma. In this disease, the hypervariable region of the clonotypic antibody represents a unique tumor antigen. Because of its consistent location, this unique tumor antigen can be readily identified with standard molecular biology techniques, and a personalized idiotype vaccine showed considerable promise in phase I/II clinical trials [50]. A randomized phase III clinical trial of a personalized idiotype vaccine has recently been completed. Patients with advanced stage follicular lymphoma that had been previously untreated were treated with standard chemotherapy. Patients with a complete response were randomly assigned to receive an idiotype vaccine conjugated to KLH, or a control vaccine without idiotype. Preliminary results demonstrated a significant prolongation in the median time to relapse (44.2 *vs.* 30.6 months; P = 0.045, hazard ratio = 1.6) [51], illustrating the potential of immune responses to unique tumor antigens.

### 4.4. The Epitope Landscape of Unique Tumor Antigens

Investigators have tried to develop high throughput strategies for the identification of unique tumor antigens [52]. Unfortunately, these strategies (differential gene expression analysis of tumor and corresponding normal tissue, in combination with acid elution of HLA molecules and mass spectrometry) are too impractical for routine use and require significant amounts of tumor tissue. Until the advent of cancer genome sequencing, there were no practical experimental techniques for the rapid and/or systematic identification of unique tumor antigens, and as such, an experimental bias may be responsible for the underrepresentation of unique tumor antigens in the scientific literature [53].

As the generation of mutations in tumor cells is continuous due to imperfect DNA replication and repair, one would predict the presence of multiple unique tumor antigens in tumor cells during tumor progression. Supporting this prediction is a recent *in silico*-based prediction analysis of the epitope landscape in breast and colorectal cancer [54]. A set of somatic mutations termed the consensus coding sequences that represent highly curated genes identified in breast and colorectal cancers [55] were analyzed for the presence of T cell epitopes to HLA-A2 using epitope prediction algorithms such as BIMAS [56]. Based on this analysis, it was predicted that individual breast and colorectal cancers accumulate an average of 10 and seven unique HLA-A*0201 epitopes, respectively. As individual cells potentially express six distinct HLA class I alleles, the total number of new epitopes was estimated to range from ∼60 to ∼42 in each breast and colorectal cancer, respectively. It should be noted that these are theoretical numbers; for example, it was not assessed in this study whether these epitopes are actually expressed by tumors, and whether the mutated proteins are processed and presented to immune cells.

Since then, several tumor genome sequencing studies have been completed that permit the search for unique tumor antigens in actual tumors. The first such study focused on the sequencing and comparative analysis of a tumor and normal genome from patients with AML for the unbiased discovery of tumor-specific somatic mutations that alter the protein-coding genes [14,57,58]. More recently, massively parallel DNA sequencing was used to sequence and compare four DNA samples (primary tumor, brain metastasis, xenograft of the primary tumor, and peripheral blood) from a patient with basal-like breast cancer [13]. Massively parallel sequencing technologies are particularly well suited to cancer genome sequencing. Breast cancer is a heterogenous disease, but genome sequencing at an almost 40× haploid coverage provides the opportunity to precisely calculate mutant allele frequencies, demonstrating genome remodeling, and unexpected similarities between the brain metastasis and xenograft [13]. We performed “epitope landscape” analyses based on the genomic analyses of primary tumor and peripheral blood DNA samples from a patient with basal-like breast cancer (Table 3). Following validation, we identified 37 candidate unique tumor antigens associated with an alteration in the amino acid sequence (nonsynonymous mutations). Amino acid sequences reflecting the mutation plus 10 flanking residues on each side were screened for binding to HLA-A2 (the breast cancer patient sequenced in these studies is HLA-A2^+^) using two prediction algorithms [52,56]. These algorithms have proven to be reliable predictors of HLA class I binding, particularly for frequently expressed alleles such as HLA-A2. Three candidate unique tumor antigens are predicted to be strong binders (predicted affinity <50 nM), while 12 are predicted to be weak binders (predicted affinity 50–500 nM). Thus, 40% of the candidate unique tumor antigens have the potential to bind HLA-A2. Of note, the HLA type of the patient under investigation is A*02, 33; B*15(71), 35; C*03, 07. We performed similar analyses to predict if candidate unique tumor antigens are capable of binding to HLA alleles other than HLA-A2. Taken together, 17 strong binders and 45 weak binders were identified for the six HLA class I alleles (data not shown). Overall, 32/37 of the candidate unique tumor antigens (86%) were predicted to bind at least one HLA allele.

## 5. Implications for Cancer Vaccines

Although cancer vaccines have been disappointing in the past [67], preliminary data from three large randomized phase III clinical trials have now demonstrated a statistically significant clinical benefit in lymphoma [68], prostate cancer [69], and melanoma [70], suggesting that cancer vaccines will have a clinical impact in these diseases in the near future. The results of tumor genome sequencing studies prompt the following questions related to the design of cancer vaccines: (1) What is the best strategy to target unique tumor antigens, a personalized vaccine approach, or an off-the-shelf vaccine approach targeting recurrent mutations? (2) Is it best to target candidate epitopes, or to design an unbiased strategy? (3) What is the best vaccine platform for targeting unique tumor antigens?

### 5.1. Personalized or Off-The-Shelf Vaccine?

The remarkable number of candidate unique tumor antigens that are predicted to bind patients' HLA class I alleles underscores the potential of an unbiased personalized vaccine approach. Given the diversity of mutations observed, the limited number of recurrent mutations present in patients' tumors, and the fact that off-the-shelf vaccines would be restricted by HLA type, we believe that relatively few cancer patients would be eligible for an off-the-shelf vaccine targeting the most common mutation(s). Even if multiple off-the-shelf vaccines were available, only a limited number of patients would be eligible. However, given the remarkable number of mutations consistently observed in cancer, it is likely that at least a subset of these mutations could be successfully targeted by a personalized vaccine approach.

### 5.2. Candidate Epitope or Unbiased Approach?

There are two conceptual strategies for creating a personalized cancer vaccine targeting unique tumor antigens: a candidate epitope approach, and an unbiased approach. The candidate epitope approach would use computer algorithms [52,56] to predict immunodominant epitopes, which could then be integrated into a personalized vaccine. Specifically targeting candidate epitopes has several advantages including ease of vaccine design and manufacture, streamlined immune monitoring, and prevention of immunodominance, a poorly understood process whereby cellular immunity is focused on one, or only a limited number of antigenic determinants, even during immune responses to complex antigens [71]. However, there are also disadvantages to a candidate epitope approach. Epitope prediction algorithms have significant limitations, particularly for less common HLA alleles, and validation of results would be costly and labor-intensive. Ultimately, it is not clear that computer algorithms or *in vitro* validation studies would be able to meaningfully predict the immunodominant antigens reliably. On balance, therefore, we believe that the unbiased approach is superior. In the unbiased approach, no attempt is made to identify the immunodominant epitopes, and all unique tumor antigens are integrated into a personalized vaccine. This approach is particularly attractive, as it has been predicted that not all unique tumor antigens are processed and presented by the immune system [54,72]. An unbiased approach, therefore, allows the immune system the opportunity to process and present the entire constellation of unique tumor antigens, maximizing the potential to successfully target the immunodominant epitope(s). An unbiased approach is also very feasible as DNA vaccines targeting multiple epitopes (polyepitope DNA vaccines) have been extensively evaluated in the infectious disease and cancer vaccine fields [73-76], taking advantage of the inherent flexibility of the DNA vaccine platform.

### 5.3. Design of a Polyepitope DNA Vaccine

The observation that direct administration of recombinant DNA can generate potent immune responses established the field of DNA vaccines in the early 1990s [77-82]. Since that time, DNA vaccines have remained an area of intense research interest, and vaccines targeting infectious disease and cancer have progressed into clinical trials. Advantages of the DNA vaccine platform include the remarkable safety profile of DNA vaccines, and the relative ease of manufacture relative to proteins and other biologics. Perhaps most important, however, is the molecular flexibility of the DNA vaccine platform, with the ability to genetically manipulate encoded antigens, and/or incorporate other genes to modulate immune response [83,84]. For instance DNA vaccines have been engineered to improve antigen expression [85-93], target dendritic cells [94,95], and/or coexpress molecular adjuvants capable of enhancing immune responses such as costimulatory molecules [96], cytokines [97-100], or chemokines [101]. For these reasons, we believe that the DNA vaccine platform is ideally suited to the clinical translation of a personalized cancer vaccine strategy.

**Figure 3.**
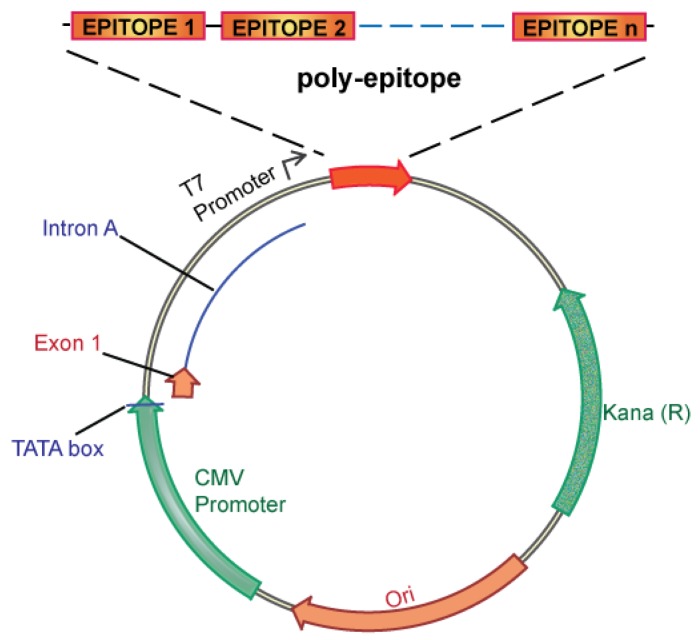
Template of a polyepitope DNA vaccine.

We propose an unbiased approach, integrating all of the candidate unique tumor antigens into a polyepitope DNA vaccine (Figure 3). Alternatively, analyzing tumor cells for expression of mutated antigens, e.g., by mRNA analysis could be implemented to select only for those mutations that are likely to be translated. This could be useful in case the number of mutations is considerably larger than the ∼60 to 42 expected mutations in breast and colon cancer, respectively. Several *in vitro* and *in vivo* studies in mice and humans have validated such a polyepitope approach, demonstrating that vaccination with peptide, viral or DNA polyepitope constructs can successfully elicit CD8^+^ T cell responses [75,102-106]. Polyepitope vaccines using plasmid DNA or viral vectors have included greater than 30 contiguous immunodominant epitopes. Quite remarkably, most epitopes appear to be successfully processed and presented from the polyepitope constructs as determined by activation of antigen-specific CD8^+^ T cells. While these earlier polyepitope vaccines employed minimal peptide epitopes, *i.e*., 9–10 amino acid in length, an argument can be made for extension of each epitope to potentially include epitopes for several different HLA alleles, and potentially also include CD4 helper epitopes. Further optimizations, which may include (1) epitope length, (2) flanking sequences and proteolytic cleavage, and (3) fusion to ubiquitin and degradation, have the potential to greatly increase the immunogenicity of polyepitope DNA vaccines.

One potential drawback of cancer vaccine is the instability of HLA expression in the tumors. However, the conventional notion that cancers downregulate and/or lose classical HLA class I expression may not be accurate in all cancers [107]. Experimental results and clinical data have demonstrated that the expression of HLA antigens can be retained in some tumors. Furthermore, some dysplastic and malignant cells can even acquire or upregulate HLA class I expression. Cancer cells are under continuous selective pressure from host's immune system (immunoediting) [26]. Therefore, the status of HLA expression on cancer cells is the results of a complex interplay between the tumor cells, the immune system, and the tumor microenvironment [107]. Nevertheless, the changes in HLA expression in cancer can profoundly affect the efficacy of any cancer immunotherapy.

### 5.4. Clinical Translation of Genome Sequencing Data into Personalized Cancer Vaccines

**Figure 4.**
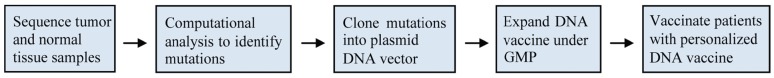
Schematic overview of the personalized cancer vaccines strategy.

The development of cancer vaccines is an area of intense investigation and considerable yet unfulfilled promise. The current paradigm is the development of cancer vaccines targeting shared tumor antigens. The dramatic evolution of cancer genome sequencing offers an attractive alternative to target unique tumor antigens. A critical challenge is how to rapidly translate genome sequencing information into personalized therapies capable of reducing cancer mortality. We argue for an innovative new paradigm: development of personalized cancer vaccines targeting unique tumor antigens identified by genome sequencing. Below is a schematic overview of how we envision personalized cancer vaccines can be created and clinically tested (Figure 4).

Cancer patients with locally/locally advanced disease will be enrolled at the time of diagnosis. Patients are to be treated with standard therapy including chemotherapy, surgery, and radiation therapy. In parallel, DNA from the primary tumor and peripheral blood will be sequenced and compared. Nonsynonymous mutations will be identified and validated. A polyepitope DNA vaccine integrating all candidate unique tumor antigens will be designed, synthesized and validated. The validated construct will be manufactured and vialed under the GMP conditions. Product release tests will be performed. After completion of standard therapy, most patients are successfully disease-free, but are at high risk of recurrence (minimal residual disease). At that time, the patients will be treated with their personalized cancer DNA vaccines.

To assess the immune response to the personalized breast cancer vaccine strategy, ELISPOT analyses and multi-parametric flow cytometry could be considered with the overall goal to determine the magnitude (ELISPOT assays) and qualitative attributes (multi-parameter flow cytometry) of vaccine-induced T cell responses. Patient-derived PBMC will be used for derivation of autologous Epstein-Barr virus immortalized lymphoblastoid B cell lines and as a source of dendritic cells and pre-immune T cells. Autologous B cell lines expressing the personalized vaccine construct can be used to stimulate T cells for ELISPOT and multi-parameter flow cytometry analysis. Importantly, the ability of vaccine-induced T cells to recognize and lyse autologous tumor can be determined using patient-derived xenografts. From each patient, T cell lines can be developed using purified CD8^+^ T cells and dendritic cells expressing the polyepitope vaccine construct. The *ex vivo* function of these T cell lines can be assessed in cytotoxicity assays using patient-derived xenografts. Altogether, these studies will provide a foundation for validation and future development of personalized breast cancer vaccines based on genome sequencing.

## 6. Conclusions

In summary, human genome sequencing has dramatically evolved of the past decade to the point where cancer genome sequences can be rapidly obtained at affordable cost. This has opened the door towards personalized medicine such as the design of vaccines against unique tumor antigens. This conceptually innovative new paradigm, coupled with the flexibility of DNA vaccine platform, represents an attractive approach to translate genome sequencing information into personalized cancer immunotherapy. We anticipate and excitedly await the development of a new generation of vaccines specifically targeting unique antigens expressed exclusively by a patient's tumor.

## Figures and Tables

**Table 1.** Summary of DNA sequencing platforms 1.

**Platform**	**Technology**	**Model**	**Length of read**	**Throughput (per day)**	**Company**
Automated Sanger sequencer	Capillary electrophoresis, BigDye^®^-terminator chemistry	3730 xl	Up to 900 bp	<3 Mb	Applied Biosystems
454	Pyrosequencing	GS FLX^+^	500–700 bp	700 Mb	454 Life Sciences (Roche)
SOLiD™	Sequencing by ligation	5500 xl	75 bp	30 Gb	Life Technologies (ABI)
Illumina	Clonal single molecule array	HiSeq2000	50–150 bp	Up to 55 Gb	Illumina, Inc.
Complete genomics	DNA nanoball array, ligation-based sequencing	N/A 2	70 bp	8.8 Gb	Complete Genomics
Ion Torrent	Hydrogen ion semiconductor	Ion 316 Chip	100 bp	100 Mb 3	Life Technologies (ABI)
HeliScope™	Imaging single nucleotide incorporation	Single Molecule Sequencer	35 bp	1 Gb	Helicos
PacBio	SMRT™ technology	PacBio *RS*	>1,000 bp	500 Mb 4	Pacific Biosciences

1 Typical performances of selected model systems are listed based on the marketing materials from each company. Actual results may vary depending on specific sample and genomic characteristics;

2 Not commercially available. Complete Genomics offers in-house sequencing services bundled with web-based data analysis;

3 Output per chip;

4 Based on *Enterobacteria phage λ* at 45 Mb/SMRT cell, 12 SMRT cells/day.

**Table 2.** Unique and shared tumor antigens.

**Characteristics**	**Unique TA**	**Shared TA**
**Expression in tumor**	single tumor	multiple tumors
**Mutation**	yes	no *
**Expression in normal tissue**	no	yes
**Risk for autoimmunity**	no	yes
**Predicted T cell affinity**	high	moderate to low
**Applicability to tumor targeting**	universal	restricted
**Resistance to immunoselection**	yes	no

* Exceptions are mutations in oncogenes/tumor suppressor genes such as K-RAS and p53.

**Table 3.** Unique tumor antigens identified through breast cancer genome sequencing and predicted binding to HLA-A2.

**Gene Symbol**	**Mutation Location 1**	**Mutation Type**	**Amino Acid Sequence 2**	**Predicted Affinity 3**	

				**mutated**	**wild type**
DDX11	12; 31122692	SNV (T > G)	QEDFMAELYRGLEAGKIGIFE	15	25
PTCH2	1; 45068225	SNV (C > G)	CHGFSHKFMHSQEELLLGGMA	26	21
PARVA	11; 12496610	insertion	SFAFELMQDGMEGLEKPKPRPE	32	18192
JAK2	9; 5040714	SNV (T > C)	QWRHDFVHGWTKVPVTHETQE	55	31
DYNC2H1	11; 102687902	SNV (G > A)	EQISKKDNTHQAHALFSLAWF	60	19004
CMV pp65	N/A	N/A	NLVPMVATV 4	N/A	60
PPPDE1	1; 242935580	SNV (A > T)	LQSCLPKEWLSPAALQSSVSQ	65	102
SHE	1; 152723308	deletion	AVFDSIPEVVHYYSLSKGQNT• 5,6	66	18757
SLC44A1	9; 107137789	SNV (G > A)	LKTLSDVQKFTEINGSALCSY	94	82
JAK2	9; 5040714	SNV (T > C)	QWRHDFVHGWTKVPVTHETQE	111	291
NALCN	13; 100688137	SNV (A > T)	VIGTTLHVYPELYHSQFTYFQ	127	1293
GP100_280_	N/A	N/A	YLEPGPVTA 4	N/A	135
GUK1	1; 226395989	SNV (C > A)	MAGSQKEEIMQPQQGVPFQES	162	331
DHDDS	1; 26646672	SNV (G > A)	FLNVCFAYTSHHEISNAVREM	186	378
MAP3K8	10; 30789749	SNV (C > T)	DLGALAGYFNLVRGLPTLEYG	216	961
DHDDS	1; 26646672	SNV (G > A)	FLNVCFAYTSHHEISNAVREM	414	889
FAM107B	10; 14603968	SNV (C > T)	ELQKVMEKRKQDQVIKQKEEE	429	949

1 Mutation location is from Ensembl build 54. The first number is the chromosome; the second indicates the first mutated nucleotide;

2 The indicated peptide sequences (21-mers) were screened for candidate epitopes of 8–11 amino acids in length. The minimal epitope with the highest predicted affinity is underlined. Amino acids that differ from the wildtype sequence are indicated in red;

3 Predicted affinity (nM) based on the NetMHC 3.2 prediction algorithm [59]. The NetMHC 3.2 server predicts binding of peptides to a number of different HLA alleles using artificial neural networks (ANNs) and weight matrices [60-62]. Affinity scores of <50 nM indicate strong binding, whereas scores between 50 and 500 nM indicate weak binding. Similar data were obtained using a second prediction algorithm [63];

4 Commonly used immunodominant peptides derived from cytomegalovirus (CMV pp65) [64] and melanoma (gp100_280_) [65] are highlighted in yellow;

5 Please note that one limitation of next-generation sequencing technologies is that it can be very difficult to identify and validate small structural variants such as insertions or deletions. Robust computer algorithms have been established for the identification of these structural variants or indels [66]. Because indels are frequently frame shift mutations, they significantly alter the amino acid sequence, and may be more likely to be recognized by the immune system;

6 • indicates stop codon.
